# Clinical Outcomes and Long-term Survivorship After Osteochondral Autologous Transfer Combined With Valgus High Tibial Osteotomy: An Analysis After 19 Years With 56 Patients

**DOI:** 10.1177/03635465241280231

**Published:** 2024-10-03

**Authors:** Yannick J. Ehmann, Thekla Esser, Romed P. Vieider, Marco-Christopher Rupp, Julian Mehl, Andreas B. Imhoff, Sebastian Siebenlist, Philipp Minzlaff

**Affiliations:** * Department of Sports Orthopaedics, Technical University of Munich, Munich, Germany; † Department of Orthopedic Sports Medicine, Orthoclinic Agatharied, Agatharied, Germany; Investigation performed at Department of Sports Orthopaedics, Technical University of Munich, Munich, Germany

**Keywords:** high tibial osteotomy, HTO, osteochondral autologous transfer, OAT, varus malalignment, osteochondral defect

## Abstract

**Background:**

Osteochondral defects of the medial femoral condyle combined with varus malalignment in young and active patients are a debilitating condition, which can result in early osteoarthritis. Osteochondral autologous transfer (OAT) combined with valgus high tibial osteotomy (HTO) might therefore be a comprehensive solution to maintain long-term knee function.

**Purpose/Hypothesis:**

The purpose of this study was to report clinical results and survivorship after combined OAT and valgus HTO for symptomatic osteochondral defects of the medial femoral condyle in the setting of varus malalignment at a long-term follow-up. It was hypothesized that undergoing combined OAT and valgus HTO would produce favorable clinical results along with a low rate of conversion to arthroplasty.

**Study Design:**

Case series; Level of evidence, 4.

**Methods:**

All patients treated between 1998 and 2008 with combined valgus HTO and OAT for deep osteochondral defects of the medial femoral condyle and concomitant varus malalignment >2° without meniscal repair/transplantation, osteoarthritis, or ligamentous instability/reconstruction were included. The survival rates of this combined procedure were evaluated. Failure was defined as conversion to knee joint arthroplasty during the follow-up period. Patient-reported outcomes were collected pre- and postoperatively, including the Lysholm score, visual analog scale score, Knee injury and Osteoarthritis Outcome Score (KOOS), Tegner Activity Scale score, and subjective level of satisfaction (scale 0-10).

**Results:**

Of 74 patients who were included for 10-year follow-up, 3 had died. A total of 15 patients were lost to follow-up, so 56 patients could be reevaluated, for a follow-up rate of nearly 80%. The mean age at surgery was 38.8 ± 9.9 years (range, 19.9-62.4 years), and the mean follow-up time was 18.9 ± 3.0 years (median, 18.8 years; range, 14.1-24.8 years). The survival rates were 87% at 10 years, 86% at 15 years, and 77% at 19 years after surgery. At final follow-up, the Lysholm score showed a mean increase of 39 points (95% CI, 25.4-50.0 points; *P* < .001) from 40 points to 79 points, representing a significant improvement. Overall, 96% of patients surpassed the minimal clinically important difference (MCID) for the Lysholm score. The visual analog scale score decreased by a mean of 4.8 points (range, 5-10 points) from 7.5 points to 2.7 points (*P* < .001), and 80% of patients surpassed the MCID. The mean Tegner Activity Scale score was 4.5 ± 1.6, and the mean KOOS subscale scores at final follow-up were as follows: Pain: 81 ± 21 (range, 19-100), Symptoms: 80 ± 22 (range, 21-100), Activities of Daily Living: 85 ± 21 (range, 18-100), Sports: 68 ± 32 (range, 0-100), and Quality of Life: 67 ± 28 (range, 0-100). Overall, 78% of the patients were satisfied with the results of the operation.

**Conclusion:**

The combination of OAT and valgus HTO presents a viable treatment option for patients affected by osteochondral defects of the medial femoral condyle and concurrent varus malalignment. A sustained and substantial improvement in clinical outcomes, significantly reduced pain severity, and a high rate of long-term survivorship can be anticipated in the long-term follow-up.

Within the spectrum of knee pathology affecting young and active patients, there is a relatively high prevalence of focal chondral and osteochondral defects within the weightbearing zone of the medial femoral area in the setting of a constitutional varus malalignment.^15,22^ Focal cartilage defects not only cause pain and functional impairment but also increase the risk of progression to early osteoarthritis, which might require the implantation of a unicondylar or total knee arthroplasty (TKA).^20,23,27^ There is a mounting body of evidence demonstrating that cartilage repair can prevent this progression and should be considered early in symptomatic patients.^10,26^

Several proven cartilage repair techniques, such as microfracture, osteochondral autologous transplantation, and autologous chondrocyte transfer, as well as single-stage cartilage repair procedures with minced cartilage, have come to the fore.^42,45^ However, osteochondral autologous transfer (OAT) is the only procedure for cartilage repair that can restore genuine hyaline cartilage in the setting of an osteochondral lesion.^43^ More precisely, OAT is a single-stage procedure that immediately restores the chondral surface while also addressing chondral and osteochondral defects by transferring the entire osteochondral unit. Several studies have proven it to be a safe and reliable technique in terms of short- and midterm results. Furthermore, OAT for defects in the medial femoral condyle has demonstrated superior results to defects in the lateral femoral condyle or patellofemoral defects.^17,19,36^

Physiologically, the alignment of the knee joint constitutes a mild varus, which slightly loads the medial compartment more than the lateral compartment.^1^ If increased load in the medial department due to varus malalignment is not corrected during the surgery, the pressure on cartilage repairs is higher and might lead to inferior results. Therefore, high tibial osteotomy (HTO) is a reliable concomitant procedure to reduce the load in the medial compartment.^14^ Combining cartilage repair and HTO has shown a superior clinical short-term outcome compared with cartilage repair alone in patients with varus malalignment.^6,13^ Combining OAT and HTO has already been proven to be a successful treatment option in patients with deep osteochondral defects in the setting of a varus malalignment at a minimum 10-year follow-up.^34^

However, a profound understanding of safety, durability, clinical outcome, and patient satisfaction—especially concerning long-term outcome—remains pivotal in discerning the optimal treatment in these patients. Similarly important is evaluating whether relatively young individuals will need future surgical interventions.^24,35^

Therefore, the aim of this study was to report clinical results and survivorship after combined OAT and valgus HTO for symptomatic osteochondral defects of the medial femoral condyle in the setting of varus malalignment at a >14-year final follow-up. It was hypothesized that undergoing combined OAT and valgus HTO would produce favorable clinical results along with a low rate of conversion to arthroplasty at long-term follow-up.

## Methods

This retrospective case series study of prospectively collected data received institutional review board ethics approval (IRB Project No. 5606/12). Patients who underwent OAT and concomitant HTO for osteochondral defects of the medial femoral condyle combined with varus malalignment between 1998 and 2008 were included in this study.^34^ This study included active individuals with deep and symptomatic high-grade osteochondral lesions (Outerbridge grade 4, equivalent to International Cartilage Regeneration & Joint Preservation Society grade 4) on the medial femoral condyle without corresponding bipolar tibial defects, as shown by magnetic resonance imaging, and a concomitant varus deformity >2°, as determined using full-leg weightbearing radiographs, with no signs of osteoarthritis based on the Kellgren-Lawrence score.^28^ Patients with previously or simultaneously performed anterior cruciate ligament or posterior cruciate ligament reconstructions or concomitant meniscal repairs/meniscal transplantations were excluded. The study population included patients previously evaluated at earlier follow-up time points.^34^

### Clinical Outcomes

The survival rate of the procedure was assessed. Failure was defined as conversion to partial or TKA during the follow-up period. Also, additional surgeries that were not a TKA were reported.

The patient-reported outcome measures (PROMs) assessed included the Lysholm score, visual analog scale (VAS) score, Knee injury and Osteoarthritis Outcome Score (KOOS) with its 5 subgroups, and Tegner Activity Scale score. Additionally, patients were asked to rate the occurrence of pain during daily activities using the following categories: “never,”“occasionally,” or “regularly.” For the evaluation of satisfaction level, a scale of 0 (not satisfied) to 10 (very satisfied) was used. The VAS and Lysholm scores were analyzed with regard to the validated minimal clinically important difference (MCID) defined in the current literature (MCID Lysholm score, 10.1; MCID VAS, 2.7) and the Patient Acceptable Symptom State (PASS) (PASS Lysholm score, 70.0, PASS VAS, 4.0).^8,25,37^

### Surgical Procedure

Each surgical procedure began with an arthroscopic assessment of the chondral condition to confirm the need for further surgery and to preclude any prohibitive concomitant irregularities. Subsequently, access to the joint was achieved through a medial parapatellar arthrotomy. The osteochondral defect in the medial femoral condyle was exposed, and its exact diameter was measured using circular templates.

The diameter and quantity of necessary autografts were determined based on the dimensions of the defect. OAT was performed using the OAT System (Arthrex Inc). The defect was prepared for transplantation using a cylindrical, hollow chisel. The cylindrical grafts were harvested from the medial and/or lateral trochlea depending on the number and size of the transferred cylinders. Plugs were inserted via a press-fit mechanism into the prepared osteochondral defect site. Harvest sites were left open, and donor-site repair was expected via natural healing processes, resulting in cancellous bone filling of the tunnels and reparative fibrocartilage coverage of the surface by marrow-derived cells.^18^

Based on the size of the defect, a variable number of grafts were transplanted to fully restore the defect while maintaining stable chondral and bony margins adjacent to the grafts.^39^ Subsequently, HTO was performed. The degree of correction was established based on the difference in chondromalacia stages observed in the medial and lateral compartments, as evaluated during the arthroscopic evaluation. If grade 4 lesions according to the Outerbridge classification were detected in the lateral compartment, no osteotomy was performed, leading to the exclusion of such cases from this series. In the cases in which grade 3 changes were present, only correction to neutral position was performed. In grade 2 lesions, a slight overcorrection of 2° of valgus was performed, while in all other cases an overcorrection of 4° of valgus was performed.

Importantly, because of its superior surgical practicability and fewer adverse effects, from 2004 onward medial open-wedge HTO as described by Hinterwimmer et al^21^ was used for varus correction instead of lateral closed-wedge HTO as described by El-Azab et al,^12^ which was used between 1998 and 2004. The surgical technique was changed with the introduction of improved locking internal fixation devices. The advantages of the new technique were decreased soft tissue dissection, avoidance of fibular osteotomy or disruption of the proximal tibiofibular joint, less risk of neurovascular injury, and the ability to more precisely adjust angular correction.

The postoperative regimen consisted of 6 weeks of nonweightbearing using crutches, without restricting range of motion. Physical therapy was carried out at least twice a week, and continuous passive motion via a motorized splint (for a minimum of 4 hours daily) was recommended for 6 to 8 weeks. This phase was followed by a gradual increase in loading, starting with 20 kg per week, until the patient's own body weight was reached.

### Statistical Analysis

Data were analyzed using SPSS Version 28.0 (IBM Corp). Continuous variables are presented as means and standard deviations. Categorical variables are presented as counts and percentages. Normally distributed continuous variables were assessed using the Shapiro-Wilk test. Nonnormally distributed continuous variables are reported as median (range). For normally distributed continuous variables, a paired *t* test was used to assess significant differences at different follow-up time points. For nonnormally distributed continuous or ordinal parameters, the Wilcoxon signed rank-test was used. For assessment of survivorship, the Kaplan-Meier method was used to estimate the probability of failure and survival as a function of time. Additionally, Cox proportional hazards models were used to estimate hazard ratios with 95% confidence intervals.

## Results

**Figure 1.**
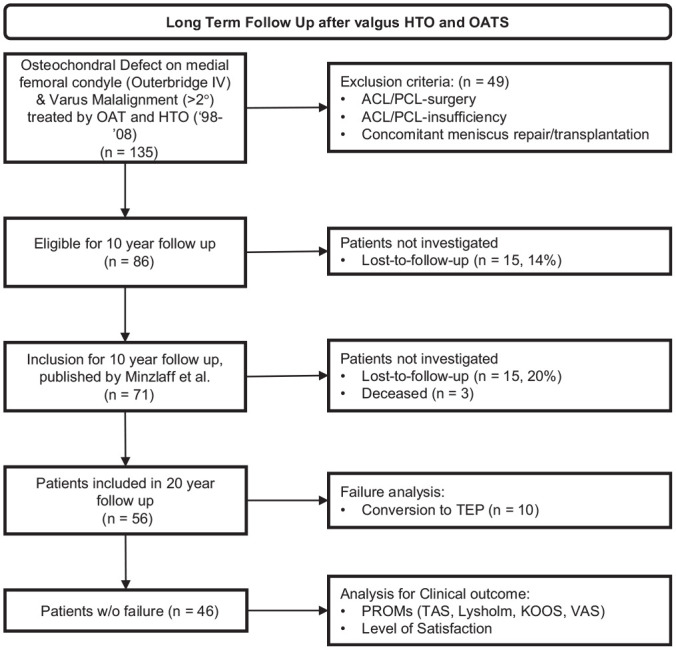
Flowchart illustrating the inclusion and exclusion process over time. ACL, anterior cruciate ligament; HTO, high tibial osteotomy; KOOS, Knee injury and Osteoarthritis Outcome Score; OAT, osteochondral autograft transfer; PCL, posterior cruciate ligament; PROM, patient-reported outcome measure; TAS, Tegner Activity Scale; TEP, total endoprosthesis; TKA, total knee arthroplasty; VAS, visual analog scale.

Of 74 patients who were included for 10-year follow-up, 3 had died. A total of 15 patients were lost to follow-up, so 56 patients could be reevaluated, for a follow-up rate of nearly 80% (Figure 1).

The remaining 56 patients (43 male, 13 female) had a mean long-term follow-up of 18.9 ± 3.0 years (median, 18.8 years; range, 14.1-24.8 years). The mean age at surgical intervention was 38.8 ± 9.9 years (range, 19.9-62.4 years), and the mean body mass index (BMI) was 28.5 ± 4.3. In 30 cases surgery was performed on the left knee, and in 26 cases on the right knee. The mean preoperative varus malalignment was 6.5°± 1.9° (range, 3°-12°). The number of transferred osteochondral plugs was 1 in 18 patients (32.1%), 2 in 23 patients (41.1%), 3 in 12 patients (21.4%), 4 in 2 patients (3.6%), and 5 in 1 patient (1.8%). Plug diameter ranged from 8 to 13 mm to fully cover the defect. The mean defect size measured intraoperatively was 21.0 ± 9.8 mm (range, 8-55 mm) in diameter. A total of 35 patients (63%) patients received previous operative treatment (Table 1).

**Table 1:** Preoperative Patient Characteristics and Descriptive Patient Data^*a*^

	Value
Age at surgery, y	38.8 ± 9.9 (19.9-62.4)
Male (n = 43)	37.1 ± 9.7 (18.5-57.3)
Female (n = 13)	40.1 ± 11.6 (19.9-62.4)
Follow-up, y	18.9 ± 3.0 (14.1-24.8)
BMI	28.5 ± 4.3 (22.2-39.4)
Preoperative varus malalignment, deg	6.5 ± 1.9 (3.0-12.0)
Correction, deg	6.9 ± 2.3 (3.0-14.0)
Size of defect, mm	21.0 ± 9.8 (8-55)
Cylinder size, mm	10.5 ± 1.5 (8-13)
No. of cylinders, median (range)	2 (1-5)

a Data are presented as mean ± SD (range) unless otherwise indicated. BMI, body mass index.

### Survivorship Analysis

**Figure 2.**
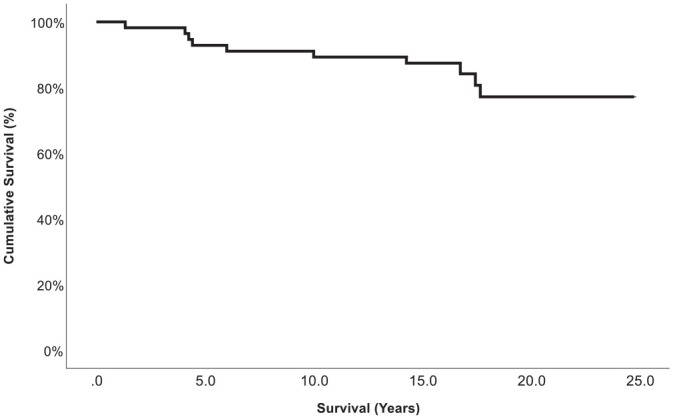
Kaplan-Meier plot showing the cumulative survival (%) of high tibial osteotomy and a concomitant osteochondral autologous transfer procedure as a function of time (years).

**Figure 3.**
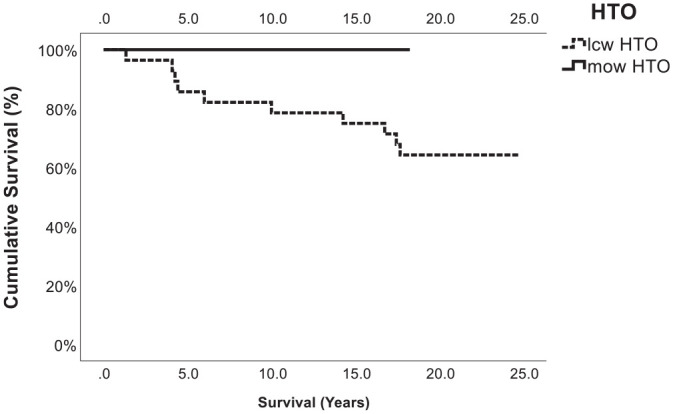
Kaplan-Meier plot showing the cumulative survival (%) of medial open-wedge high tibial osteotomy (HTO) versus lateral closed-wedge HTO and a concomitant osteochondral autologous transfer procedure as a function of time (years). The graph shows that no failure (conversion to total knee arthroplasty) occurred in the medial open-wedge group, which was statistically significant (*P =* .004). lcw, lateral closed-wedge; mow, medial open-wedge.

After the index procedure, the mean survival rates were 87.4% at 10 years, 85.8% at 15 years, and 77.2% at 18.9 ± 3.0 years (Figure 2). In 10 patients, with a mean age of 47.3 ± 9.0 years (range, 33.3-62.4 years), the combination of OAT and HTO failed after a mean time of 9.6 ± 6.4 years (range, 1.3-17.7 years) and resulted in a conversion to a TKA. Within the failure group (7 male, 3 female), 3 patients received knee surgery (1 lateral release, 2 medial meniscus partial resection) before the index procedure. The mean varus correction in this group was 9.2°± 2.9° (range, 6.0°-14°), and the mean size of the OAT cylinder was 10.7 ± 0.5 mm (range, 10-11 mm). All osteotomies in these 10 patients were performed using a closed-wedge technique (Figure 3, Table 2).

**Table 2:** Baseline Characteristics of the Failures Defined as Conversion to TKA (n = 10)^*a*^

Patient ID	Age at Surgery, y	BMI	Varus Malalignment, deg	Cylinder Size, mm	Correction, deg	Mow or Lcw	No. of Cylinders	Time Until Failure, y
1	50	30	4	10	6	Lcw	1	4
2	42	26	6	11	6	Lcw	2	17
3	56	26	4	10	8	Lcw	2	18
4	33	32	10	11	14	Lcw	5	1
5	62	27	6	11	10	Lcw	2	6
6	39	29	5	11	6	Lcw	1	4
7	49	32	8	11	12	Lcw	3	4
8	49	33	7	10	10	Lcw	4	10
9	53	28	6	11	12	Lcw	2	14
10	38	25	6	11	8	Lcw	1	17

a BMI, body mass index; Lcw, lateral closed-wedge HTO; Mow, medial open-wedge HTO.

**Figure 4.**
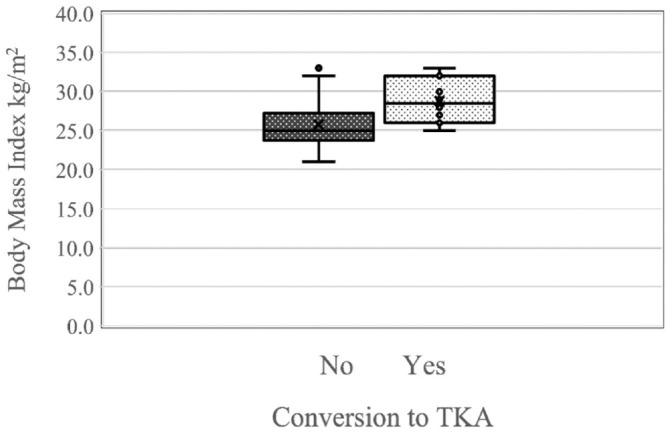
Box plot showing the effect of the body mass index (BMI) on the failure rate, defined as conversion to total knee arthroplasty (TKA). Each 1-point increase in BMI corresponded to an estimated risk increment (hazard ratio) for failure of 1.6 (95% CI, 1.06-2.35), which was statistically significant (*P* = .023).

When analyzing the risk of failure, BMI at the time of the index surgery was a significant influence. Each incremental rise in BMI by 1 point corresponded to a 1.6-fold increase in the estimated risk of failure (95% CI, 1.06-2.35; *P* = .023) (Figure 4).

Age, varus correction in degrees, size of the OAT cylinders, and number of cylinders were not associated with a statistically significant increased risk of conversion to TKA.

Four patients required additional surgery, excluding those who required a TKA. Three patients had additional microfracture on a different site in the knee, and 1 patient had arthroscopic surgery for a hematoma.

### Clinical Outcomes

**Figure 5.**
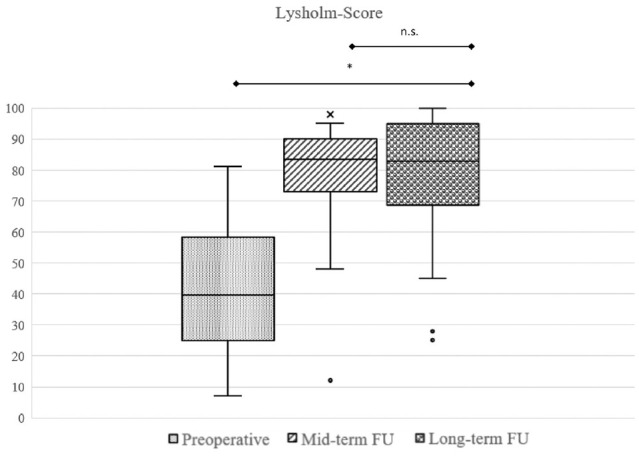
Box plot showing that regarding the difference in Lysholm score from preoperatively to long-term follow-up (FU), there was a significant improvement. *P = .004. There was no significant (n.s.) change between midterm follow-up and long-term follow-up (*P* = .230).

**Figure 6. fig6-03635465241280231:**
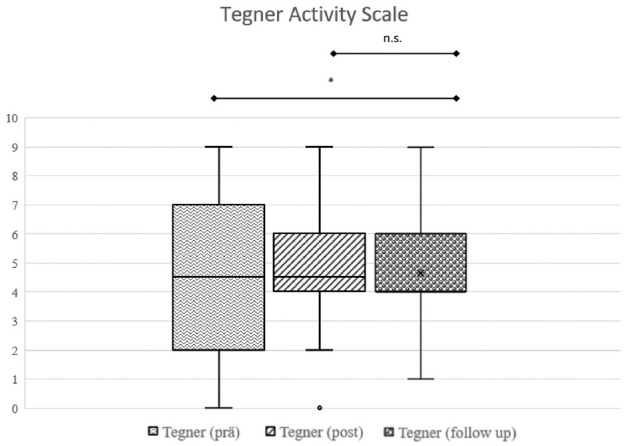
Box plot showing that regarding the difference in Tegner Activity Scale score from preoperatively to long-term follow-up, there was a significant improvement. *P = .029. There was no significant (n.s.) change between midterm follow-up and long-term follow-up (*P =* .21). prăOP, preoperative.

The mean Lysholm score at long-term follow-up of patients who did not receive arthroplasty during the follow-up time (n = 46) was 79.0 ± 19.2 (range, 25-100). Comparison of preoperative scores with PROMs at final follow-up revealed statistically significant improvement in all PROMs. There were no significant changes between the 10-year follow-up and final follow-up (Figures 5 [Fig fig6-03635465241280231]-7; for all clinical results, see Table 3). Overall, 96% (n = 44) surpassed the MCID for the Lysholm score and 72% (n = 33) reached the PASS for the Lysholm score (Figure 8).

**Figure 7.**
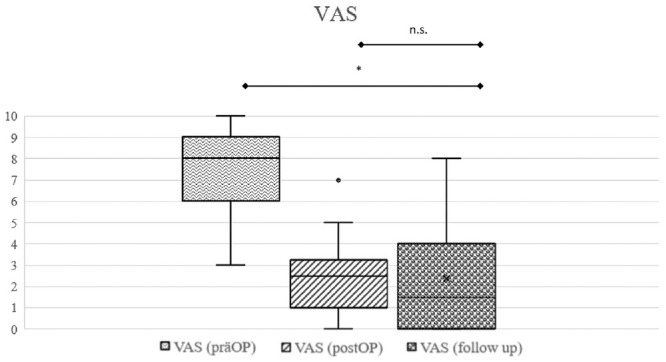
Box plot showing that regarding the difference in the visual analog scale (VAS) score from preoperatively to long-term follow-up, there was a significant improvement. *P = .003. There was no significant change between midterm follow-up and long-term follow-up (*P =* .754). prăOP, preoperative.

**Table 3:** PROMs Over Time Show Significant Improvement of Long-term Follow-up Against Preoperative Values^*a*^

	Preoperative	Midterm Follow-up	Long-term Follow-up	Preoperative vs Long-term Follow-up *P* Value	Midterm Follow-up vs Long-term Follow-up *P* Value
VAS score	7.51 ± 2.1	2.2 ± 1.6	2.3 ± 2.6	**.001**	.691
Lysholm score	41.8 ± 23.1	77.5 ± 17.3	77.6 ± 20.0	.**001**	.728
TAS score	4.3 ± 2.7	4.5 ± 2.7	4.5 ± 1.6	.354	.800
KOOS Pain		85.0 ± 13.9	81.4 ± 21.9		.504
KOOS Symptoms		79.7 ± 16.9	79.5 ± 21.7		.784
KOOS ADL		91.1 ± 12.8	84.5 ± 2.7		**.021**
KOOS Sports		66.4 ± 25.9	67.9 ± 31.9		.702
KOOS QoL		57.5 ± 22.7	67.5 ± 27.9		.**024**

a Data are presented as mean ± SD. Bold *P* values indicate statistical significance. ADL, Activities of Daily Living; KOOS, Knee injury and Osteoarthritis Outcome Score; PROM, patient-reported outcome measure; QoL, Quality of Life; TAS, Tegner Activity Scale; VAS, visual analog scale.

**Figure 8.**
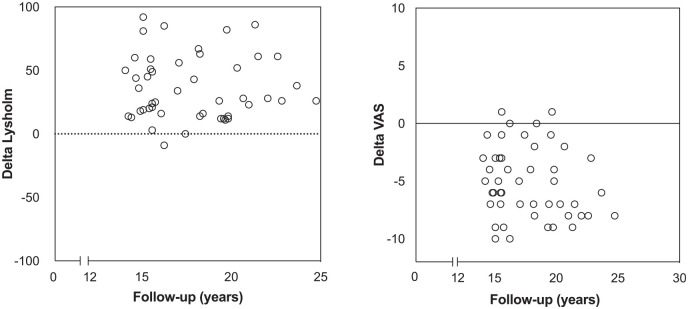
Scatterplots showing the change in Lysholm score and visual analog scale (VAS) score over time from preoperatively to long-term follow-up.

The VAS score decreased by a mean of 5.2 points (range, 5-10 points) from preoperatively to final follow-up, from 7.5 points to 2.3 points. Overall, 80% (n = 37) surpassed the MCID for the VAS score and 76% (n = 36) reached the PASS for the VAS score (Figure 8).

Of all patients, 61% reported a rating ≥9 on a satisfaction scale of 0 (not satisfied) to 10 (very satisfied). Evaluation of pain revealed that 50% (n = 23) of patients occasionally experienced pain and 22% (n = 10) did not feel any pain in their everyday life. Daily pain medication intake in everyday life was reported as follows: never (83%, n = 38), occasionally (13%, n = 6), and regularly (4%, n = 2). Occasional intake of pain medication before sport was reported by 28% (n = 13), and 72% (n = 33) denied taking any pain medication before sports activities.

## Discussion

The most important finding of this study was that there was a 77% survival rate of surgery across an observation span of 14 to 24 years for OAT and concomitant HTO among relatively young and active patients with osteochondral lesions in the medial femoral condyle and varus knee malalignment. On longitudinal assessment, there was a significant improvement regarding PROMs and pain between preoperative and the long-term assessment. Therefore, the combined treatment with a cartilage repair technique such as OAT as well as an alignment corrective procedure such as HTO appears efficacious in sustaining long-term knee function with a high rate of survival.

The rate of conversion to TKA showed favorable outcomes, with rates of 12% at the 10-year mark, 14% at 15 years, and 23% at final follow-up. Thus, this complex surgical procedure enables most patients with significant osteochondral damage of the medial femoral condyle and varus malalignment to avert the transition to TKA. Although previous studies have demonstrated this finding in short- and midterm follow-ups, ours is the first report with a comparable and longer follow-up time.^3,34^

Moreover, in our analysis of risk factors associated with surgical failure, we identified an elevated BMI as a statistically significant determinant of increased risk of surgical failure. These findings align with the current literature, which demonstrates an adverse effect of higher BMI on the outcomes of HTO and cartilage therapy interventions.^7,11,30,44^ Clinicians should consider this information to carefully select patients who will benefit from this surgical intervention. Patients who required TKA conversion after 10 years were predominantly characterized by advanced age and higher BMI and had previous knee surgical interventions, indicating that those are risk factors for failure in the first 10 years.^34^ At final follow-up, no significant effect could be shown. This might indicate that those risk factors lead to early failure but in long-term follow-up pose no significant threat of failure. However, the risk factors for failure should be further investigated.

On investigating the risk factors associated with surgical failure, a notable observation emerged: all instances of failure were attributed to patients who had undergone lateral closed-wedge HTO procedures before 2004, whereas no patient treated with medial open-wedge HTO experienced failure. Several plausible explanations for this discrepancy may be considered. First, a pivotal alteration in the treatment approach coincided with the introduction of enhanced locking internal fixation devices. These innovations ushered in several advantages, including diminished soft tissue dissection, avoidance of fibular osteotomy or disruption of the proximal tibiofibular joint, reduced risk of neurovascular injury, and enhanced precision in angular correction.^21^ Second, the increasing caseload over time likely contributed to improved standardization and refinements in surgical techniques.^5^

Regarding the additional surgeries that were not TKAs, the 3 microfractures performed on other parts of the knee joint should not be considered as failures because of the new nature of the problem. The arthroscopy performed for a hematoma can be considered as a minor complication, but the patient showed good results in the long-term follow-up.

The analysis of clinical outcomes in our study reveals noteworthy findings, characterized by a substantial improvement in PROMs. Utilized PROMs included the Lysholm score, VAS score, KOOS subscale scores, and patient satisfaction. Great portions of the collective surpassed the MCID and reached the PASS for the VAS and Lysholm scores, showing that a relevant reduction in pain and a relevant increase of knee function can be achieved at final follow-up. This achievement is particularly remarkable when considering the duration of the follow-up period, which encompassed the natural aging process of the patients. Furthermore, the study cohort originally comprised a relatively young and highly active population, for whom knee function was paramount. These outcomes align with the existing literature that has reported similar improvements in PROMs for HTO, OAT, and other cartilage repair treatments in isolation.^7,30,44^ The literature has shown that a high return-to-sports rate and an activity level comparable to the patient's 1-year preoperative state can be expected in young and active patients 10 years after combined OAT and valgus HTO. This seems to be relevant in clinical practice when planning, indicating, and performing these complex procedures.^33^ Importantly, the present study uniquely demonstrates such improvements in the context of a combined HTO and OAT treatment approach.

Additionally, the results of PROMs and patient satisfaction demonstrated no significant decline when comparing the midterm follow-up with the long-term follow-up. Even though the incidence of surgical failures increased during this interval, the clinical outcomes of nonfailures remained stable. This phenomenon emphasizes the enduring efficacy of the investigated surgical combination technique, which not only delivers favorable 10-year results but also sustains these benefits at final follow-up.

The good clinical results also indicate that when combining OAT with HTO, the donor-site morbidity of the OAT does not have a severe negative effect on the clinical outcome. The medial trochlea has been shown to be an excellent donor site with regard to the radius of the curvature and undergoes less contact stress but is a noticeably smaller area than its lateral counterpart, which limits available graft harvesting.^2,16,32^ Our findings challenge previous concerns about the safety, donor-site morbidity, and invasiveness of the OAT.^31,41^

For osteochondral lesions, autologous chondrocyte implantation (ACI) combined with autologous trabecular bone grafting might be an option.^38^ Nonetheless, irrespective of the modality, this intervention typically necessitates a 2-step process, which is potentially cumbersome for patients. When confronted with more profound osteochondral defects, it becomes evident that ACI shows limitations in comparison with OAT.^29^

In recent years, the minced cartilage technique, a 1-step procedure, has become more popular. The first studies for chondral lesions show equivalent outcomes compared with ACI in the short term with outstanding long-term evaluations.^45^ Furthermore, no data about its combination with autologous bone grafting in terms of osteochondral lesions exist.

Biological cartilage procedures are generally used for younger patients, while knee arthroplasties are reserved for older patients. Resurfacing implants are currently discussed as a solution in this treatment gap, showing good 10-year survival outcomes.^9^

For medial compartment osteoarthritic appearances, the efficacy of valgus HTO has been established, as it yields solid outcomes and survival indices.^3,7,46^ Nevertheless, the corpus of data showing the efficacy of concomitant cartilage repair and unloading osteotomies remains sparse. In 2010, Sterett et al^47^ conducted a survivorship analysis encompassing microfracture and HTO in 106 knees of patients, with a mean age of 52 years, confronting medial chondral degeneration alongside varus misalignment. Their research echoes our findings in outcomes; however, our cohort predominantly included medial femoral osteochondral lesions, thereby supporting our predilection for OAT as the primary cartilage repair strategy.

Recent literature investigating outcomes after the combination of neutralizing HTO and matrix-induced ACI has shown good to excellent results in the long-term follow-up, potentially delaying osteoarthritis.^7,30,40^ Valgus HTO was performed to mitigate graft overburdening attributable to inherent varus malalignment. It is paramount to acknowledge that cartilage restoration endeavors predominantly target younger cohorts, mirroring our study population.^4^ Furthermore, by aiming for a joint salvation, the intervention can potentially postpone or negate the necessity for TKA.

In summary, the present study supports the premise that an integrated surgical approach, encompassing OAT in combination with HTO, offers a promising therapeutic trajectory for younger, active patients grappling with pronounced osteochondral defects in the medial femoral condyle and coexisting varus knee malalignment. For cases of medial compartment arthritis, valgus HTO has demonstrated its value, yielding good results and high survival rates.^3,30,46^

To the best of our knowledge, the present study is the first to specifically investigate long-term results over 15 years and survivor rates after combining OAT and valgus HTO. The survivor rate was high compared with isolated HTO procedures for unicompartmental osteoarthritis and similar to the results of microfracture combined with HTO.^46^

The findings from this study hold substantial clinical significance. In the management of physically active individuals evaluated with deep and symptomatic high-grade osteochondral lesions located on the medial femoral condyle and concomitant varus malalignment, a definitive gold standard has not yet been established. The outcomes reported in this investigation contribute valuable insights to the discourse surrounding potential therapeutic strategies and provide a basis for discussing the anticipated clinical and long-term survival outcomes. This holds particular importance given that a substantial proportion of patients can delay the implantation of a TKA. It is demonstrable that, even in cases involving deep and symptomatic high-grade osteochondral lesions on the medial femoral condyle with associated varus malalignment, the restoration of satisfactory knee function remains a feasible and attainable objective.

However, this study has several limitations. First, this study is limited by the absence of a control group and its retrospective nature. We cannot conclusively determine whether the OAT procedure, the unloading HTO, or their combination led to the final outcome. To further address the potential bias of confounding factors, a potential prospective, multicentric study could gather further insights to limit the effect of those potential confounding factors on the results. It cannot be finally determined whether an unloading osteotomy is necessary for patients with significant malalignment undergoing OAT and vice versa. Second, this study does not provide data regarding the tolerable amount of varus deviation in cases of osteochondral resurfacing. Third, this study included both closed-wedge and open-wedge osteotomies. Even though both procedures unload the affected compartment, this results in a heterogeneous study population.

## Conclusion

The combination of OAT and valgus HTO presents a viable treatment option for patients affected by osteochondral defects of the medial femoral condyle and concurrent varus malalignment. A sustained and substantial improvement in clinical outcomes, significantly reduced pain severity, and a high rate of long-term survivorship can be anticipated in the long-term follow-up.
